# Pure Cultivation in Vivo of Vaccine Virus Free from Bacteria

**Published:** 1915-11

**Authors:** 


**Pure Cultivation in Vivo of Vaccine Virus Free from Bacteria.** Hideyo Noguchi (Jour. Exp. Med., Vol. XXI, No. 6) reports a method of cultivating vaccine virus free from bacteria, and at a lessened expense. The preparations by the skin method on calves, even though carried out under strict aseptic precautions, have a distinct bacterial content. This necessitates a "ripening" process for about three months to remove the bacteria, and during this process the activity of the virus is reduced. Furthermore the perfect bacteria-free virus is never produced in very active form. The methods of preparation advocated in this paper as a result of experimentation, consist in the cultivation by injection into the testicle of rabbits. The virus is propagated in this way practically pure and free from skin contamination and capable of indefinite transfer from one animal to another. The initial cost of the finished product is lessened, as 1000 cc. of the vaccine can be made from forty rabbits or four calves. Furthermore, epidemics among cattle, such as foot and mouth disease, often close the market to calves. No disease is yet known which need be feared with respect to rabbits. Nor is it necessary to keep them under quarantine any length of time to insure their freedom from infection. The further discovery was made that the maximum multiplication of the virus occurs the 4th or 5th day and diminishes after the 8th day. The vaccinal processes in the skin, cornea and testicle are practically identical when the skin or testicular strains of virus are used, also the skin lesions produced in the calf and in the human with the two strains are identical. The same excellent results were obtained in the experimental testicular inoculations of bulls. He believes this method superior to the skin inoculations, but subject to the same disadvantages from quarantined cattle. He finally concludes: "Pure strains of testicular virus are readily produced, and once secured may be propagated in a pure state in rabbits or bulls without difficulty and with economy. The pure strains thus obtained should supply an ideal form of virus for employment in the vaccination of human beings."—St. Paul Med. Jour.

